# Retraction

**DOI:** 10.1111/jcmm.16031

**Published:** 2021-01-04

S100A9 gene silencing inhibits the release of pro‐inflammatory cytokines by blocking the IL‐17 signalling pathway in mice with acute pancreatitis. Dong‐Mei Wu, Shan Wang, Min Shen, Yong‐Jian Wang, Bo Zhang, Zi‐Qi Wu, Jun Lu and Yuan‐Lin Zheng. J Cell Mol Med. 2018; 22: 2378–2389

The above article from Journal of Cellular and Molecular Medicine, published online on 14 February 2018 on Wiley Online Library (wileyonlinelibrary.com) in Volume 22, pp. 2378–2389, has been retracted by agreement between the authors, the journal Editor‐in‐Chief and John Wiley & Sons Ltd. The retraction has been agreed following an investigation based on allegations raised by a third party. The authors asked to retract this manuscript as raw experimental data were not accessible anymore and thus the validity of the overall results could not be confirmed.

